# Design and Optimize the Performance of Self-Powered Photodetector Based on PbS/TiS_3_ Heterostructure by SCAPS-1D

**DOI:** 10.3390/nano12030325

**Published:** 2022-01-20

**Authors:** Huizhen Yao, Lai Liu

**Affiliations:** Key Laboratory of Instrumentation Science and Dynamic Measurement, Ministry of Education, School of Instrument and Electronics, North University of China, Taiyuan 030051, China; huizhenyao@126.com

**Keywords:** simulation, TiS_3_/PbS, heterostructure, photodetector, SCAPS-1D

## Abstract

Titanium trisulphide (TiS_3_) has been widely used in the field of optoelectronics owing to its superb optical and electronic characteristics. In this work, a self-powered photodetector using bulk PbS/TiS_3_ p-n heterojunction is numerically investigated and analyzed by a Solar Cell Capacitance Simulator in one-Dimension (SCAPS-1D) software. The energy bands, electron-holes generation or recombination rate, current density-voltage (J-V), and spectral response properties have been investigated by SCAPS-1D. To improve the performance of photodetectors, the influence of thickness, shallow acceptor or donor density, and defect density are investigated. By optimization, the optimal thickness of the TiS_3_ layer and PbS layer are determined to be 2.5 μm and 700 nm, respectively. The density of the superior shallow acceptor (donor) is 10^15^ (10^22^) cm^−3^. High quality TiS_3_ film is required with the defect density of about 10^14^ cm^−3^. For the PbS layer, the maximum defect density is 10^17^ cm^−3^. As a result, the photodetector based on the heterojunction with optimal parameters exhibits a good photoresponse from 300 nm to 1300 nm. Under the air mass 1.5 global tilt (AM 1.5G) illuminations, the optimal short-circuit current reaches 35.57 mA/cm^2^ and the open circuit voltage is about 870 mV. The responsivity (R) and a detectivity (D*) of the simulated photodetector are 0.36 A W^−^^1^ and 3.9 × 10^13^ Jones, respectively. The simulation result provides a promising research direction to further broaden the TiS_3_-based optoelectronic device.

## 1. Introduction

Photodetectors that directly convert light into electrical signals have been developed for numerous applications, including medical diagnosis, aviation, target recognition, missile warning, and other fields [1,2,3,4,5,6,7]. Recently, self-powered photodetectors which can realize light detection without an external power supply have aroused a great deal of interest. The self-powered devices can work independently because of the photoelectric effect based on p–n or Schottky junction under illumination from light sources [8]. The built-in electric field existing in effective heterojunction between different materials will function as a driving force for high efficiency photogenerated carriers’ separation and produce continuous photocurrent. Photodetectors with self-powered behaviors based on p–n junction exhibit outstanding photoelectric performance, such as high response speed, large linear region, and low noise, and have achieved significant progresses [9].

Titanium trisulphide (TiS_3_) with a monoclinic structure is an n-type semiconducting material which has a direct optical bandgap of 1.0 eV [10,11]. Theoretically, TiS_3_ will be a potential candidate substitution to silicon, micro or nanostructured, due to its exceptional carrier mobility (as high as ~10^4^ cm^2^ V^−1^ s^−1^), high anisotropy, high optical absorption coefficient, and high chemical stability in the open-air [12,13]. Typically, the TiS_3_ nanoribbon material has been successfully obtained in laboratory by sulfuration of Ti film. The unique optoelectronics properties of TiS_3_ nanostructure make it wildly useful in the fields of cathodes in batteries [14,15], hydrogen storage [16,17], thermoelectric energy conversion devices [18,19,20], and optoelectronics applications [21,22,23,24]. Niu et al. have developed a mixed-dimensionality TiS_3_/Si n–p heterostructure broadband photodetector via staking an n-type TiS_3_ nanoribbon onto p-type silicon substrate. The photoresponse of the device strongly depends on the polarization direction of the illumination. The high responsivity and on/off ratio of the TiS_3_/Si device were ascribed to the improvement in charge separation coming from the coupling effect of TiS_3_ nanoribbon and Si substrate [25]. Frisenda et al. have fabricated TiS_3_-based nanoribbon photodetectors by the dielectrophoresis method between two gold electrodes. The photodetector can work efficiently in the visible region and possesses a responsivity of 3.8 mA/W [26]. Huang et al. synthesized TiS_3_ nanoribbon array film on Ti-coated glass-carbon substrate by using a chemical vapor transport method. The vertically grown TiS_3_ film with moderate S_2_^2−^ vacancies exhibits a long electron diffusion length for collecting electrons efficiently and an outstandingly high photocurrent density of 15.35 mA/cm^2^ was achieved at 1.4 V versus using reversible hydrogen electrode [27]. The TiS_3_ film has been proven as an excellent photoanode material. However, there are few reports concerning the self-powered photodetector using the TiS_3_ film.

In this work, a self-powered PbS/TiS_3_ p–n heterojunction film photodetector is numerically investigated and analyzed by one-Dimension software SCAPS-1D. By numerically modeling, the impact of thickness, defect density, and shallow acceptor or donor density on the performance of photodetectors were investigated. Under standard AM 1.5G illuminations, the achieved responsivity value is 0.36 A W^−1^ and the detectivity value is 3.9 × 10^13^ Jones of the photodetectors. The photodetector based on the heterojunction with optimal parameters exhibits a broad photoresponse in the UV-visible and near-infrared light region. The simulation result provides a promising research direction to further broaden the TiS_3_-based optoelectronic device.

## 2. Numerical Simulation and Device Structure

The numerical simulation software used in this work is SCAPS-1D (V3.3.07), developed by the Department of Electronics and Information Systems of the Gent University (Ghent, Belgium) [28,29]. The software has been extensively used for simulating the thin-film solar cells to explore the electrical and optical properties, as well as the physics involved. As per previous reports, the simulated results from SCAPS have a good agreement with the experimental results [30,31]. In recent years, a number of research works based on SCAPS-1D software explored its applications in finding highly efficient photovoltaic devices [32,33,34,35,36,37]. Fundamentally, SCAPS-1D solves three sets of equations, Poisson’s equation, hole continuity, and electron continuity under the constraint of boundary conditions. These three equations are shown below [38,39,40]:

(1) $$\frac{\partial^{2}\varphi }{\partial x^{2}}+\frac{q}{\varepsilon }[p(x)-n(x)-\rho_{n}+\rho_{p}-N_{A}+N_{D}]=0$$

(2) $$\frac{1}{q}\frac{dJ_{p}}{dx}=G_{\mathrm{op}}(x)-R(x)$$

(3) $$\frac{1}{q}\frac{dJ_{n}}{dx}=G_{\mathrm{op}}(x)+R(x)$$

where φ shows the electrostatic potential, ε is the dielectric constant, and q is the electron charge. $N_{A}$ is acceptor type and $N_{D}$ is donor type density, respectively. p(n) is hole (electron) concentration. $\rho_{p}$ ($\rho_{n}$) is hole (electron) distribution. $J_{p}$ is the current densities of the hole and $J_{n}$ is the current densities of the electron, respectively. $G_{\mathrm{op}}$ designates the optical generation rate and R is the net recombination including direct and indirect recombination. All of these parameters are the function of the position coordinate x.

**Figure 1:**
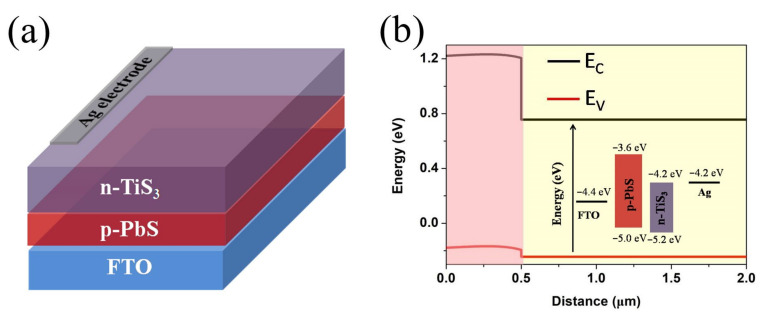
(**a**) Schematic n-TiS_3_/p-PbS heterostructure photodetector, (**b**) energy band scheme of PbS/TiS_3_ heterojunction.

The numerical modeling is an important step to understand the physical properties of, and to realize, the highly efficient photoelectronic device. The narrow band gap TiS_3_ layer acts as an absorber. The fluorine-doped tin oxide (FTO) layer is employed as a transparent conductive oxide layer. Figure 1a shows the diagrammatic drawing of the FTO/ PbS/TiS_3_/Ag thin-film heterojunction architecture photodetector. The PbS/TiS_3_ heterojunctions are constructed in the designed device. The energy band scheme for the PbS/TiS_3_ heterojunction thin-film photodetector is shown in Figure 1b. It is clearly observed that the conduction band of the PbS layer is about 0.6 eV higher compared with that of the TiS_3_ layer. The conduction band offset would promote photo-generated electrons towards the Ag electrode. Furthermore, the valence band maximum of the TiS_3_ layer is very close to that of the PbS. The estimated valance band offset is ~0.2 eV at the PbS/TiS_3_ interface, which would promote photo-generated holes’ transport to the FTO substrate.

The physical parameters of PbS and TiS_3_ layers used in this simulation are shown in Table 1. All these parameters are from previous reports and theories [12,41,42,43]. The approximate thermal velocity of electrons and holes in PbS and TiS_3_ semiconductor at room temperature is set at 10^7^ cm/s for simplifying the numerical calculation. The surface recombination velocity of both electrons and holes at the FTO or Ag electrode is assumed to be 10^7^ cm/s. The capture cross-sections of both the electron and hole are fixed at 10^−14^ cm^2^. The interface defect parameters used in the PbS/TiS_3_ heterojunction device simulation was 10^12^ cm^−3^. AM 1.5G illuminations is used in all of our tests to optimize the simulation investigation, using 1000 W/m^2^ from the PbS layer side. Photoresponsivity (R) and photodetectivity (D*) are important parameters for assessing the performance of a device and evaluate the detector sensitivity. It is assumed that the shot noise from the dark current is the primary source of total noise, R and D* are given as [44,45]:

(4) $$R=\frac{I_{\mathrm{light}}}{P_{in}S}$$

(5) $$D^{*}=\frac{RS^{1/2}}{{\left(2eI_{d}\right)}^{1/2}}$$

where $P_{in}$ is the incident light intensity, *S* represents the effective area of the device, and *e* is the elementary charge (*e* = 1.60 × 10^−19^ C).

## 3. Results and Discussion

### 3.1. Influence of p-PbS and n-TiS_3_ Layer Thickness on Device Performance

**Figure 2:**
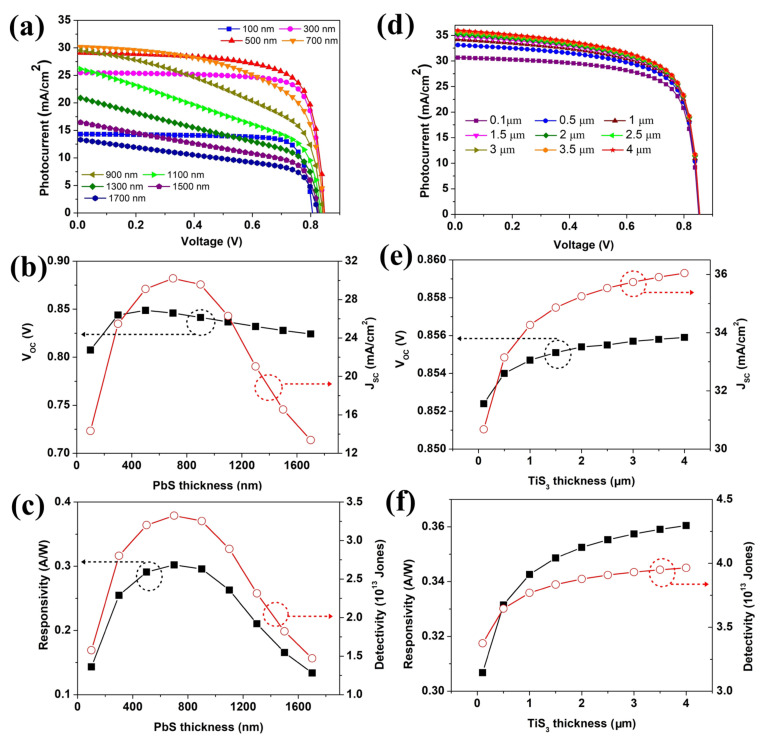
(**a**) J–V characteristic curves for varying the PbS layer thickness, (**b**) V_OC_ and J_SC_ variation, (**c**) R and D* variation with respect to the thickness of the PbS layer, (**d**) J–V characteristic curves for varying the TiS_3_ layer thickness, (**e**) V_OC_ and J_SC_ variation, (**f**) R and D* variation with respect to the thickness of the TiS_3_ layer.

The thicknesses of n-TiS_3_ and p-PbS layers are a key parameter to determine the performance of the detector. Optimizing the factor is in favor of obtaining optimal device performance. Figure 2 depicts the effect of the PbS and TiS_3_ layer thickness on the suggested photodetector performance. The PbS layer thickness was modified between 0.1–1.7 μm, while keeping the thickness of the TiS_3_ layer constant at 0.5 μm. As shown in Figure 2a,b, with the thickness of the PbS layer increasing from 0.1 μm to 0.7 μm, the short circuit photocurrent (J_SC_) obviously increased from 14.33 to 30.21 mA/cm^2^. This is attributed to the fact that the ultra-thin PbS layer leads to a large leakage current. However, as the thickness continues to rise to 900 nm, the J_SC_ begins to decrease. When the thickness of the PbS layer was 1.7 μm, the J_SC_ sharply reduced to 13.21 mA/cm^2^. This resulted from a large number of photons being absorbed by the PbS layer and, thus, a smaller number of photons being able to reach the junction between PbS and TiS_3_, which, in turn, reduced the generation of photogenerated carriers. Figure 2b shows that the variation trend of open circuit voltage (V_OC_) is similar to that of J_SC_. The appropriate thickness of the PbS layer means a higher carriers concentration, which can expand the depleted region of TiS_3_ and enhance the performance. When the thickness of the PbS layer was 0.7 μm, the responsitivity and detectivity were 0.3 A/W and 3.3 × 10^13^ Jones (shown in Figure 2c), respectively. The results indicate that the optimum thickness of the PbS layer is 0.7 μm.

To investigate the effect of TiS_3_ layer thickness, the simulation study was carried out with a thickness range from 0.1 μm to 4 μm as displayed in Figure 2d–f. It has been observed that the V_OC_ and J_SC_ of the simulated device were enhanced with increasing thickness of the TiS_3_ layer. When the thickness of the TiS_3_ layer was 0.1 μm, the photocurrent was 30.68 mA/cm^2^. The thin TiS_3_ layer could not fully absorb the incoming light resulting in low photocurrent while almost all of the photogenerated electron-hole could reach the corresponding electrode. As the thickness of the TiS_3_ layer rises, more photons are captured, resulting in a rise in J_SC_. The photocurrent increased to 35.53 mA/cm^2^ with the thickness of the TiS_3_ layer at 2.5 μm. Nevertheless, there was no significant change in the performance parameters when continuing to increase the thickness, resulting from the light absorption being saturated. The propagation path for the photo-generated carriers is long, leading to an increasing carrier recombination rate in the inner of TiS_3_ layer. The simulated device can produce a highly efficient performance when the TiS_3_ layer thickness is equal to 2.5 μm. The J–V characteristic curves for varying the PbS layer thickness with the constant optimized TiS_3_ layer at 2.5 μm are also given in Figure S1 (Supporting Information). The tendency is similar to that in Figure 2a, which verifies our conclusion. The responsivity and detectivity are 0.36 A/W and 3.9 × 10^13^ Jones (shown in Figure 2f), respectively.

### 3.2. Influence of Doping Concentration of p-PbS Layer and n-TiS_3_ Layer

**Figure 3:**
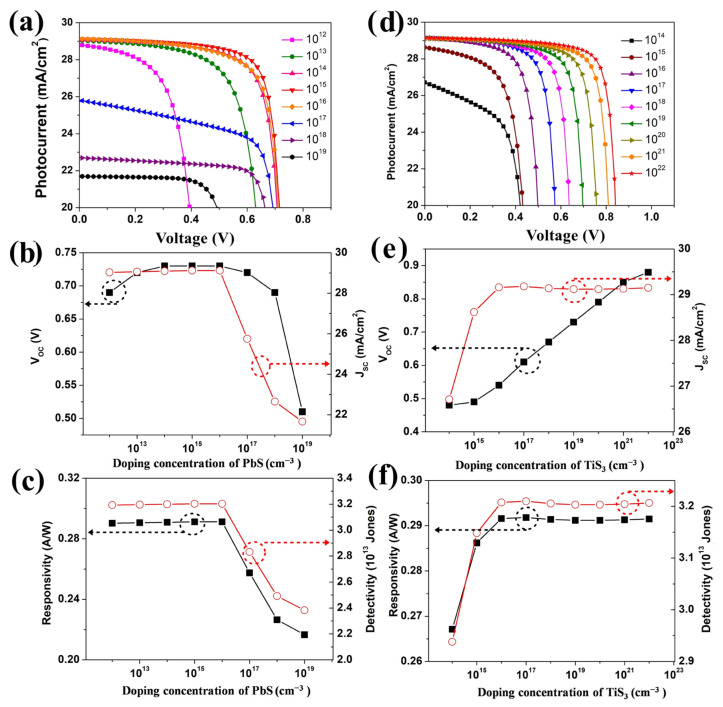
(**a**) J–V characteristic curves, (**b**) V_OC_ and J_SC_ variation, (**c**) R and D* variation for varying the PbS shallow acceptor density, (**d**) J–V characteristic curves, (**e**) V_OC_ and J_SC_ variation, (**f**) R and D* variation for varying the TiS_3_ shallow donor density.

Shallow acceptor density (*N_A_*) plays an important role in improving the performance of photodetectors. In the simulation study, the doping concentration of the PbS layer was varied from 10^12^ cm^−3^ to 10^19^ cm^−3^, while other parameters remained the same. It is shown in Figure 3a,b that the V_OC_ and J_SC_ improves with the concentration of the PbS carrier concentration rising but below 10^15^ cm^−3^, indicating that the minority charge carrier recombination was reducing. When the doping density of the PbS layer continually increased to 10^17^ cm^−3^, the overall performance of the photodetector including J_SC_, V_OC_, responsitivity, and detectivity were quenched enormously due to the increased carriers recombination, as shown in Figure S2 (Supporting Information). It is observed in Figure 3c that the maximum responsivity and detectivity are 0.29 A/W and 3.2 × 10^13^ Jones when the acceptor density is at 10^15^ cm^−3^. The results suggest that the proper doping of the PbS layer results in a more efficient performance. As shown in Figure 3d,e, the donor density (*N_D_*) of the TiS_3_ layer is ranging from 10^14^ to 10^22^ cm^−3^. It can be observed that all the performances of the simulated photodetector were enhanced with the increasing doping density of the TiS_3_ layer. It is concluded that the high doping density results in a large built-in potential at the PbS/TiS_3_ interface. Consequently, the photo-generated carrier recombination is observably inhibited. In the numerical study, the doping concentration of 10^22^ cm^−3^ is chosen to obtain the best responsivity and detectivity (as shown in Figure 3f) of the designed photodetector.

### 3.3. Influence of the Concentration of Defect Density

**Figure 4:**
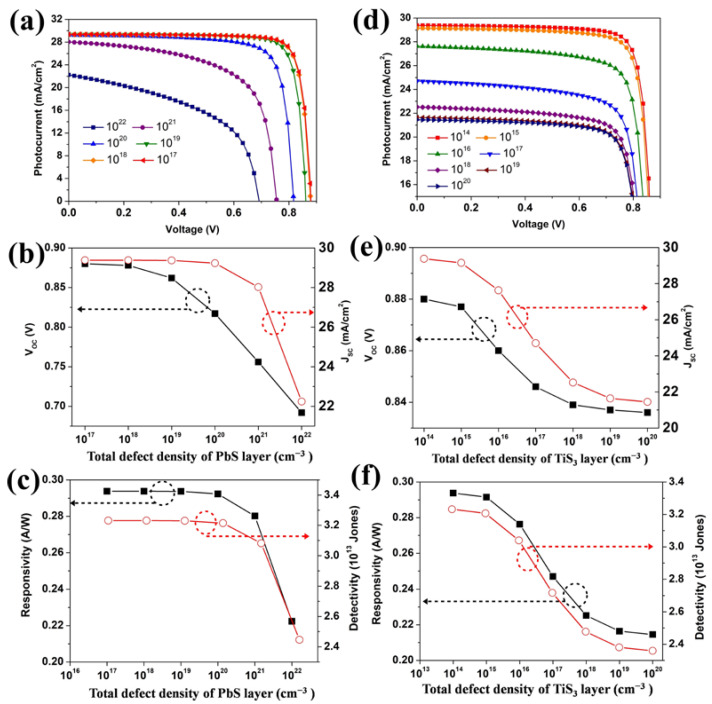
(**a**) J–V characteristic curves for varying the PbS defect density, (**b**) V_OC_ and J_SC_ variation, (**c**) R and D* variation with respect to the PbS defect density of the PbS layer, (**d**) J–V characteristic curves for varying the TiS_3_ defect density, (**e**) V_OC_ and J_SC_ variation, (**f**) R and D* variation with respect to the defect density of the TiS_3_ layer.

The performance of the device is also dependent on the defect density of each layer. The increase in defect density results in more photo-generated carrier recombination, which seriously reduces the efficiency of the device. In the study, the defect density of the PbS layer and TiS_3_ layer are varied in the range of 10^1^^2^–10^22^ cm^−3^ and 10^1^^2^–10^20^ cm^−3^, respectively. When the defect density of PbS was set from 10^12^ to 10^17,^ shown in Figure S3 (Supporting Information), the photoelectric performance of the simulated photodetector had little change. It is seen from Figure 4a,b that, given a continuous augment in the defect density of the PbS layer, V_OC_ and J_SC_ are degraded. When the defect density of the PbS layer increased to 10^22^ cm^−3^, the J_SC_ reduced to 22.24 mA/cm^2^ and the corresponding responsivity quenched to 0.22 A/W, as shown in Figure 4c. This is attributed to the raised carrier recombination rate with the localized energy levels created by the defects. The results show that only a mass concentration of defects in the PbS layer quenched the performance of the device. The optimal defect density of the PbS layer is ranged from 10^12^ to 10^17^. As shown in Figure S4 (Supporting Information), when the defect density of the TiS_3_ is ranged from 10^12^ to 10^14^, the photocurrent is kept at around 29.38 mA/cm^2^. When the defect density was magnified from 10^14^ cm^−3^ to 10^20^ cm^−3^, shown in Figure 4d,e, J_SC_ varied from 29.38 mA/cm^2^ to 21.45 mA/cm^2^. It is observed from Figure 4f that responsivity and detectivity had a similar downtrend. The optimal responsivity and detectivity are 0.29 A/W and 3.2 × 10^13^ Jones, when the defect density of the PbS layer and TiS_3_ layer are ranged from 10^12^ cm^−3^ to 10^17^ cm^−3^ and from 10^12^ to 10^14^ cm^−3^.

### 3.4. Self-Powered n-TiS_3_/p-PbS Heterostructure Photodetector

**Figure 5:**
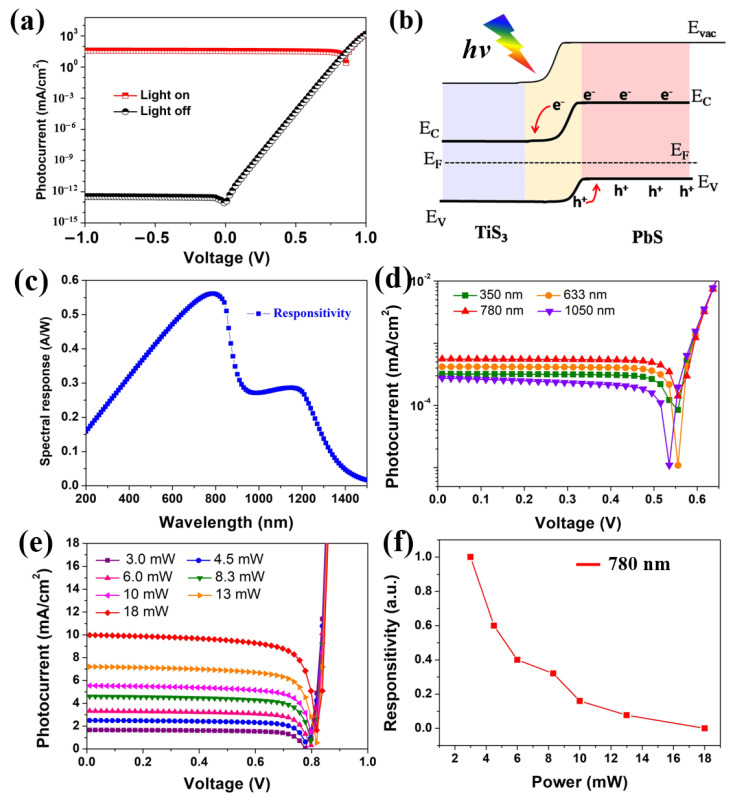
(**a**) I–V curves of the simulated photodetector with and without light illumination, (**b**) schematic band diagram after contacting, (**c**) responsivity with different illumination wavelengths at a power of 100 mW/cm^2^, (**d**) I−V characteristics of simulated PbS/TiS_3_ under different wavelengths of illumination and (**e**) illuminated with a 780 nm wavelength at different powers, (**f**) dependence of responsitivity of the photodetector versus the light illumination power.

Through simulated optimizing, the thickness of the TiS_3_ layer and PbS layer are 2.5 μm and 700 nm, respectively. The density of the acceptor or donor is set at 10^15^ or 10^22^ cm^−3^. The high quality TiS_3_ film is required to have a defect density of about 10^14^ cm^−3^. For the PbS layer, the maximum defect density is 10^17^ cm^−3^. The optoelectronic performances of the simulated n-TiS_3_/p-PbS heterostructure devices in dark and AM 1.5G standard illuminations are shown in Figure 5a. In the dark, the photodetector displays a typical rectifying I–V characteristic due to the heterostructure formed at the interface between the n-TiS_3_ and the p-PbS. Under AM 1.5G illuminations, an enhanced photocurrent is observed. The photocurrent of the simulated photodetector after majorization is 35.57 mA/cm^2^. The optimal photoresponsivity of the proposed heterostructure device is about 0.36 A W^−1^ and the corresponding detectivity is 3.9 × 10^13^ Jones, which is comparable with the photodetector based on nanostructured silicon [46,47]. The ratio of light and dark current (*I*_light_/*I*_dark_) is about 10^14^ at bias voltages of 0 V. This phenomenon suggests that the photodetector can be triggered by itself. The built-in electric field which formed at the TiS_3_ and PbS interface can separate the photogenerated carriers even at zero bias. The photoelectrical properties displayed by the TiS_3_–PbS device can be clarified by the band scheme of the PbS and TiS_3_ materials, as displayed in Figure 5b. The band gap energies of TiS_3_ and PbS semiconductors are about 1.0 and 1.4 eV, respectively. As for the insulate layer, the different inherent nature leads to a different position of the Fermi levels (Supporting Information Figure S5). The electrons at the interface will be transported from the high level to the lower and, in turn, produce a potential difference at the contact interface. This phenomenon is displayed in the band scheme by the bending of the conduction and the valence band at the interface (as shown in Figure 5b). The rectifying I−V characteristics and the photovoltaic effect noticed in the proposed photodetector resulted from the type-II band mechanism. Figure 5c shows the photoresponsivity of the simulated PbS/TiS_3_ photodetector device. The device shows different light response characteristics under different light wavelengths. A distinct responsivity ranged from the UV to the near-infrared region is observed, which indicates the excellent broadband performance of the photodetector. With 780 nm illumination, the photodetector shows superior responsibility, as shown in Figure 5d. Figure 5e shows the I−V characteristics of the simulated device upon illumination (with 780 nm of wavelength) with enhanced optical power. It is noted that the photocurrent enhances monotonically with the augmenting light power density. This phenomenon can be a result of the growing number of photogenerated carriers as the light intensity increases. On the contrary, the responsivity decreases (Figure 5f). The R values of the photodetectors are large under low light power density, indicating that the simulated photodetectors are very sensitive to weak light.

## 4. Conclusions

In summary, a self-powered PbS/TiS_3_ heterostructure photodetector is numerically investigated. Herein, the PbS/TiS_3_ photodetector is modeled and optimized using SCAPS-1D software. The important parameters, including the energy bands, electron-holes generation or recombination rate, current density–voltage (J–V), and spectral response properties of the proposed device, have been explored. The influence of thickness, shallow acceptor or donor density, and defect density are also investigated. As a result, the photodetector based on the heterojunction with optimal parameters exhibits a good photoresponse from 300 nm to 1300 nm. Under AM 1.5G illuminations, the optimal short-circuit current reaches 35.57 mA/cm^2^ and the open circuit voltage is about 870 mV. The responsivity and a detectivity of the simulated photodetector are 0.36 A W^−1^ and 3.9 × 10^13^ Jones, respectively. The simulation result paves a promising way for further broadening the applicability of the TiS_3_-based optoelectronic device.

## Figures and Tables

**Table 1:** Parameters set for the simulation of TiS_3_-based photodetector.

Properties	FTO	PbS	TiS_3_
Thickness (nm)	300	200	500
Band gap (eV)	3.6	1.4	1.0
Electron affinity (eV)	4.0	4.35	4.8
Dielectric permittivity (relative)	9.0	10	9.98
Electron thermal velocity (cm/s)	1 × 10^7^	1 × 10^7^	1 × 10^7^
Hole thermal velocity (cm/s)	1 × 10^7^	1 × 10^7^	1 × 10^7^
CB effective DOS (cm^−3^)	2.2 × 10^18^	1 × 10^18^	1 × 10^18^
VB effective DOS (cm^−3^)	1.8 × 10^19^	1 × 10^18^	1.8 × 10^19^
Donor density N_D_ (cm^−3^)	1 × 10^17^	0	1 × 10^18^
Acceptor density N_A_ (cm^−3^)	0	1 × 10^17^	0
Electron Mobility (cm^2^/Vs)	100	50	200
Hole mobility (cm^2^/Vs)	25	10	94

## Supplementary Materials

The following supporting information can be downloaded at: https://www.mdpi.com/article/10.3390/nano12030325/s1, Figure S1: J–V characteristic curves for varying the PbS layer thickness with constant optimized TiS_3_ layer at 2.5 μm, Figure S2: Total recombination of photo carriers in the TiS_3_-based photodetector with varied shallow acceptor density of PbS layer, Figure S3: J–V characteristic curves for varying the PbS defect density from 10^12^ to 10^17^ cm^−3^, Figure S4: J–V characteristic curves for varying the TiS_3_ defect density from 10^12^ to 10^14^ cm^−3^, Figure S5: Schematic band diagram before TiS_3_ and PbS contacting.
