# Supplementary material for: Design and Optimize the Performance of Self-Powered Photodetector Based on PbS/TiS3 Heterostructure by SCAPS-1D
Source: Nanomaterials (Basel). 2022 Jan 20;12(3):325. doi: 10.3390/nano12030325 (PMC8838530; doi:10.3390/nano12030325)
Supplement: Supplementary file 1 [file nanomaterials-12-00325-s001.zip › nanomaterials-1545864-supplementary.pdf]

nanomaterials

Supporting Information

Design and Optimize the Performance of Self-Powered Photodetector Based on PbS/TiS_3 Heterostructure by SCAPS-1D

Huizhen Yao and Lai Liu *

Key Laboratory of Instrumentation Science and Dynamic Measurement, Ministry of Education, School of Instrument and Electronics, North University of China, Taiyuan 030051, China; huizhenyao@126.com
* Correspondence: liulaiopto@163.com

Supplementary Materials:

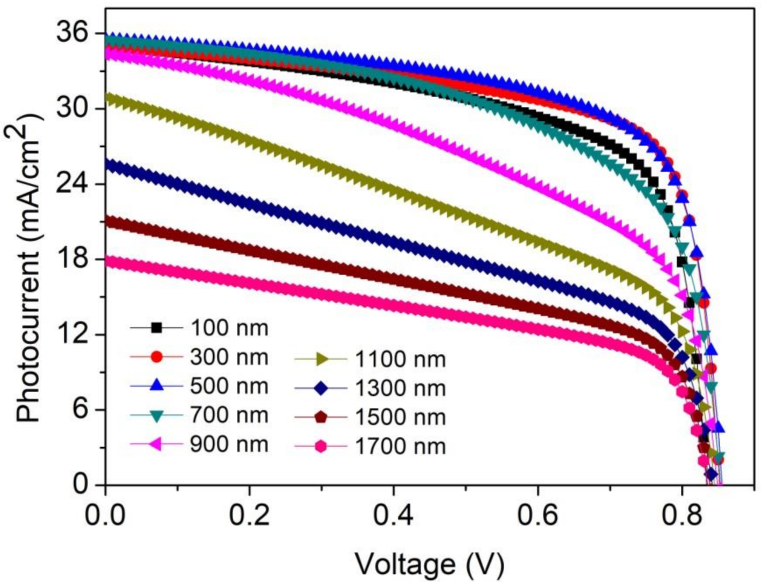

Figure S1. J–V characteristic curves for varying the PbS layer thickness with constant optimized TiS_3 layer at 2.5 μm.

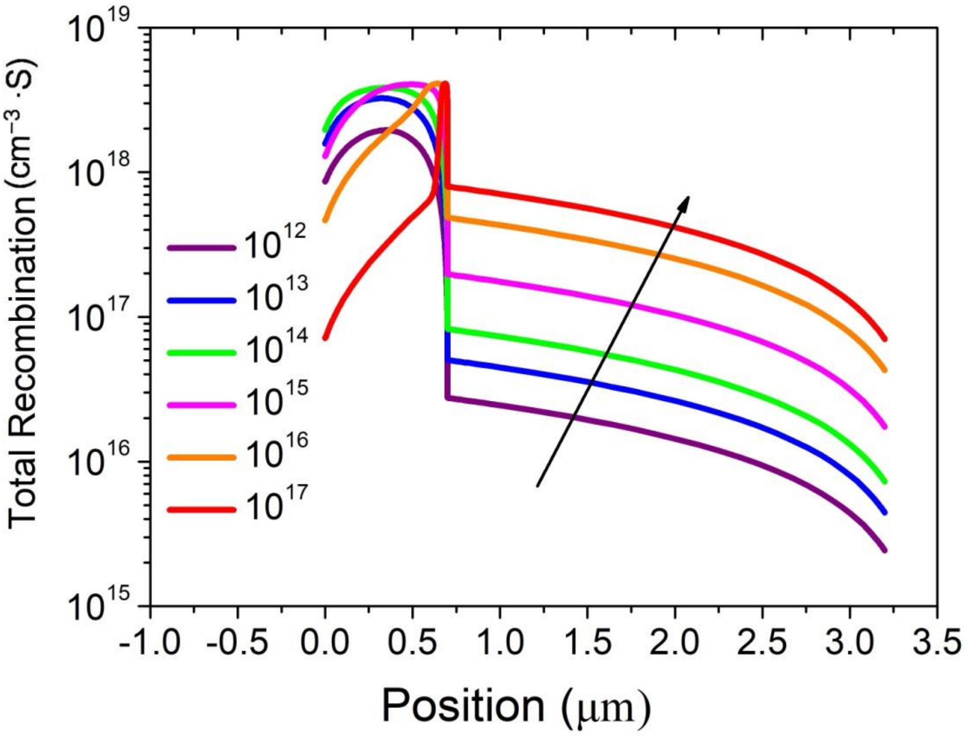

Figure S2. Total recombination of photo carriers in the TiS_3-base photodetector with varied shallow acceptor density of PbS layer.

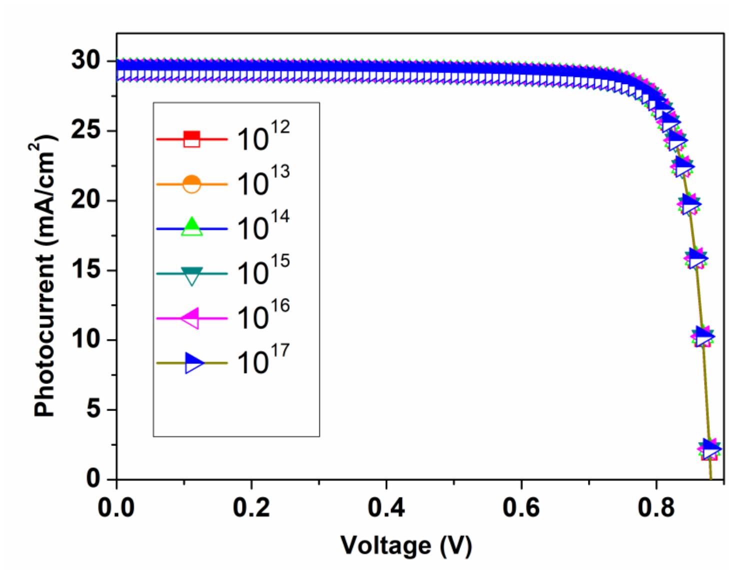

Figure S3. J–V characteristic curves for varying the PbS defect density from 10^{12} to 10^{17} cm^{-3}.

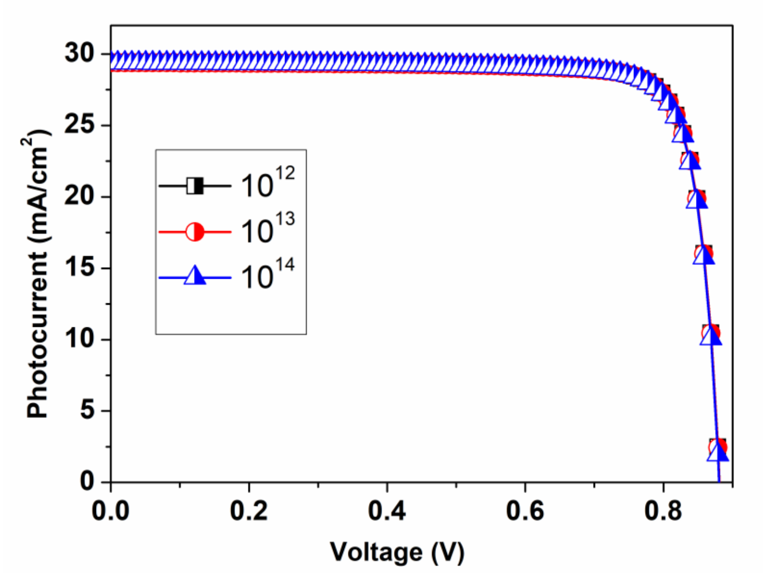

Figure S4. J–V characteristic curves for varying the TiS_3 defect density from 10^{12} to 10^{14} cm^{-3}.

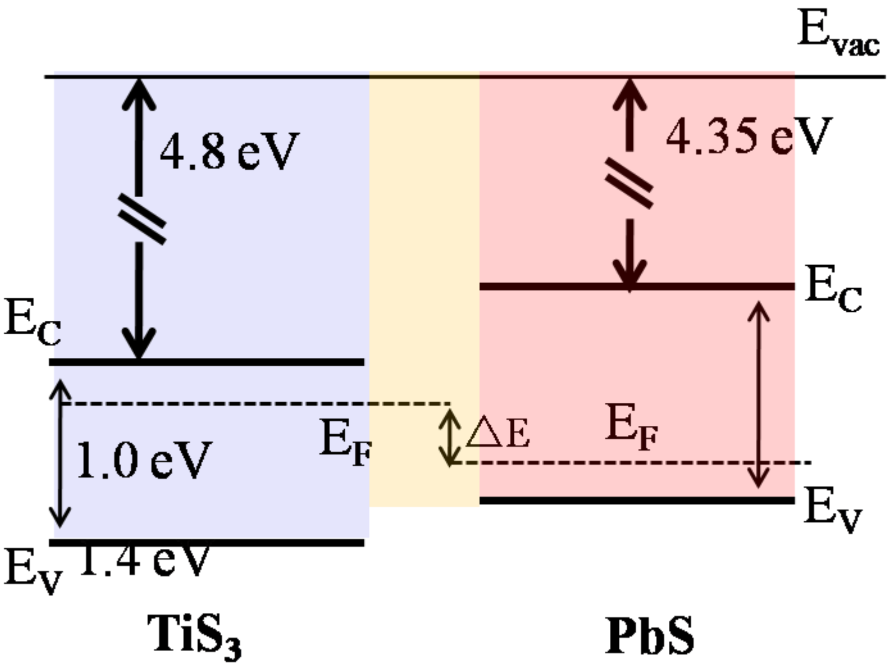

Figure S5. Schematic band diagram before TiS_3 and PbS contacting.
